# Investigation of Salivary Function and Oral Microbiota of Radiation Caries-Free People with Nasopharyngeal Carcinoma

**DOI:** 10.1371/journal.pone.0123137

**Published:** 2015-04-10

**Authors:** Jingyang Zhang, Hongling Liu, Xue Liang, Min Zhang, Renke Wang, Guang Peng, Jiyao Li

**Affiliations:** 1 State Key Laboratory of Oral Diseases, West China Hospital of Stomatology, Sichuan University, Chengdu, 610041, China; 2 Department of Operative Dentistry and Endodontics, West China School of Stomatology, Sichuan University, Chengdu, 610041, China; 3 Radiation Physics Center, Cancer Center, West China Hospital, Sichuan University, Chengdu, 610041, China; National Institute of Dental and Craniofacial Research, UNITED STATES

## Abstract

Radiation caries have been reported to be correlated with radiotherapy-induced destruction of salivary function and changes in oral microbiota. There have been no published reports detailing patients who have remained radiation caries-free following radiotherapy for nasopharyngeal carcinoma. The aim of this study was to investigate the relationship between salivary function, oral microbiota and the absence of radiation caries. Twelve radiation caries-free patients and nine patients exhibiting radiation caries following irradiated nasopharyngeal carcinoma were selected. V40, the dose at which the volume of the contralateral parotid gland receives more than 40 Gy, was recorded. Stimulated saliva flow rate, pH values and buffering capacity were examined to assess salivary function. Stimulated saliva was used for molecular profiling by Denaturing Gradient Gel Electrophoresis. Mutans streptococci and *Lactobacilli* in saliva were also cultivated. There were no significant differences in V40 between radiation caries-free individuals and those with radiation caries. Compared with normal values, the radiation caries-free group had significantly decreased simulated saliva flow rate, while there were no significant differences in the saliva pH value and buffering capacity. Similar results were observed in the radiation caries group. There was no statistical difference in microbial diversity, composition and log CFU counts in cultivation from the radiation caries-free group and the radiation caries group. Eleven genera were detected in these two groups, among which *Streptococcus* spp. and *Neisseria* spp. had the highest distribution. Our results suggest that changes in salivary function and in salivary microbiota do not explain the absence of radiation caries in radiation caries-free individuals.

## Introduction

Radiotherapy (RT) is a major treatment method used in nasopharyngeal carcinoma (NPC) patients [1]. However, the most common and severe sequela of RT is radiation caries [2–7]. Previous research has shown that nearly 90% of irradiated NPC patients suffered from radiation caries [4, 5, 8]. Radiation caries is a rapidly developing and highly destructive form of tooth decay, and tends to develop as quickly as four weeks after completion of RT [6]. In addition, it is worth noting that patients are at highest risk of developing radiation caries in the first year following RT [9, 10]. Destruction of salivary function and shift of oral microbiota have been reported as potential factors of the disease[11]. However, due to the potential multifactorial nature of the disease, there are still open question regarding other potential factors [6, 10, 12, 13]. Most of the prior research has focused on irradiated patients within the first year of RT [14, 15]. Little research has investigated irradiated patients more than one year after the completion of RT.

There are many reports on the correlation between the flow rate of saliva and radiation caries [16–19]. It is known that a significant decrease of saliva flow rate and the destruction of salivary function are commonly observed after RT [20, 21]. It has been reported that 85% of irradiated patients with a stimulated saliva flow rate of less than 0.5ml/min develop at least one carious lesion per year. The higher the rate of saliva secretion is, the lower the risk of radiation caries [21]. As a result, the practice of RT has adopted new techniques to reduce the decrease in salivary function. In recent years, intensity-modulated radiotherapy (IMRT), which can deliver higher RT doses to target volumes while sparing critical structures such as the parotid glands and submandibular glands, has been widely used in RT of NPC patients [22, 23]. It has been demonstrated that the effect on salivary function brought on IMRT is significantly less destructive than ordinary RT [22, 24]. Still, the incidence of radiation caries did not show significant decrease [25, 26].

Additionally, changes in the oral microbiota in oral cavity is considered to be yet another potential factor in the development of radiation caries [27]. In patients who have undergone RT, a higher number of acidogenic and aciduric flora was detected by traditional culturing techniques after RT than before [7, 14, 28]. In recent years, the rapid development of advanced molecular biology methods has provided important insights into the oral microbiome. Using polymerase chain reaction-denaturing gradient gel electrophoresis (PCR-DGGE) fingerprinting, a culture-independent molecular fingerprinting technique, the predominant bacterial species can be rapidly assessed [29]. When this technique was applied to the oral microbiota of patients undergoing RT, a shift in the major taxa of oral microorganisms was observed [30]. Some researchers have also found that the total strepcocucci increased with decreased saliva flow rate [2]. However, it has also been reported that there is no significant shift of diversity, stability and microbial composition detected by PCR-DGGE within the first six months after IMRT [3]. Taken together, the current data do not show a clear relationship between the high risk of developing radiation caries and the changes in the oral microbiota within the first year following RT.

Most of the existing research on this topic are based on data from irradiated patients who finished their RT within the first year [3, 30, 31]. To our knowledge, there is currently no research focused on NPC patients who never developed new dental caries more than one year after IMRT. Thus, the purpose of this study is to access potential factors from radiation caries-free individuals. We hypothesize that the salivary function and the salivary microbiota are correlated to the absence of radiation caries after one year of RT.

## Methods

### Ethics statement

Approval for this study was received from the ethics committee of the West China Dental Hospital of Sichuan University (2011017). Informed and written consents were obtained from the patients who were recruited into the study from July 2012 to March 2014. All study subjects gave their voluntary written consent for taking part in this research.

### Study design and subject selection

The present study employed a cross-sectional case-cohort design. Irradiated patients in the study were all NPC patients from West China Hospital in Sichuan Province, China, who were treated using the Precise X-ray accelerator (Sweden), and the plans of RT including dose at which the volume of parotid received were designed through ADAC Pinnacle3-9.0 (Philips, Netherland). All of patients had completed their IMRT at least one year before taking part in this study. Inclusion and exclusion criteria are displayed in Table 1.

**Table 1:** Admission criteria.

Radiation caries-free (RCF) group	
Inclusion criteria	Exclusion criteria
-having at least 28 teeth in the oral cavity	-Receiving antibiotics within 3 months before the study
-finish RT at least one year	-priodontitis
-over eighteen years	-smoker
-having no newly decay of teeth after RT	
-Free of systemic disease(including systemic infection)	
-written informed consent	
**Radiation caries (RC) group**	
**Inclusion criteria**	**Exclusion criteria**
-age, sex, after RT period comparable with RCF individuals	-receiving antibiotics within 3 months before the study
-having newly decay of teeth after RT(DMFT≥6)	-priodontitis
-free of systemic disease(including systemic infection)	-smoker
-written informed consent	

### History record

The bilateral size of the parotid gland, dose value of RT and V40, the volume of the contralateral parotid gland receiving more than 40Gy, were taken from the dental record held in the West China Dental Hospital of Sichuan University. V40 was shown to be the best predictive dose-value variable for salivary flow recovery [32].

### Oral examination

Before sampling, a questionnaire was issued to the subjects on their oral hygiene habits, dietary habits, clinical application of fluoride products, use of artificial saliva, and their subjective feeling of oral dryness [15]. Dental examinations for caries popularity were all carried out by the same research fellow, who adopted the WHO criteria for the diagnosis and coding of dental caries. Dental caries were scored by the sum of decayed, missing and filled teeth (DMFT). The plaque index, defined by Sillness and Loe [1964], was also recorded.

### Measurement of salivary function

All saliva sample collection were conducted in the morning. Each subject was asked not to eat or drink for at least 2 hours prior to the sample collection. After resting for 5 minutes with no talking, subjects were asked to chew on a piece of paraffin wax for 30 seconds and expectorate directly into a graduated 50 mL sample collection tube on ice within 5 minutes[33]. The pH value of the stimulated saliva was measured using a pH meter (Metrohm632, Herisau, Switzerland) [28]. Buffering capacity was measured by the dip-slide technique (CRT bacteria, Ivo-clar Vivadent, Germany) according to Köhler and Bratthall[1997]and classified following the manufacturer’s instructions[34].

### Microbial sampling

Twenty-one stimulated saliva samples were collected in total for genetic fingerprinting and culture. Samples were immediately transferred on ice to a microbiology laboratory (State Key Laboratory of Oral Diseases, West China Hospital of Stomatology, Sichuan University).

### Microbial cultivation

Samples were shaken (30 s) and diluted (10^-1^ to 10^-5^-fold). Brain heart infusion (BHI, Difco) with defibrinated 5% sheep blood agar, Mitis-Salivarius-Bacitracin (MSB) agar [35] and Rogosa-SL agar (4080; Difco) [28, 35, 36] were used for microbial culture. The diluted samples (10 μl) were plated on media that selected for Mutans Streptococcus (MS) and *Lactobacilli* (LB). Plates were incubated under 80% nitrogen, 10% carbon dioxide, and 10% hydrogen for 48 h, and colonies on each culture plate were counted. Colonies were collected and MS and LB were positively identified by Gram staining, microscopy, and sequencing. Colonies on each culture plate were manually counted, while the logarithm of the CFU values was calculated to estimate the distribution of both targeted microorganism in plaque and saliva, as well as for statistical analysis.

### DNA extraction and PCR assay

Total bacterial genomic DNA was extracted from clinical samples using QIAamp DNA Micro Kit (Qiagen) using the manufacturer’s instructions. The concentration and purity of the extracted DNA was measured using a Nanodrop 2000 spectrometer (Thermo Fisher Scientific, Wilmington, USA). An approximately 300-bp internal fragment of the 16S rRNA gene was amplified using the universal primer set, Bac1 (5′-CGC CCG CCG CGC CCC GCG CCC GTC CCGCCG CCC CCG CCC GAC TAC GTG CCA GCA GCC-3′) and Bac2 (5′-GGACTA CCA GGGTAT CTA ATC C-3′)[37]. Each 50-μl PCR tube containeds 100 ng purified genomic DNA, 40 pmol of each primer, 200 μM of each dNTP, 4.0mM MgCl_2_, 5μl 10× PCR buffer, and 2.5 U Taq DNA polymerase (Invitrogen, California, USA). Cycling conditions were 94°C denaturation for 3 min, followed by 30 cycles of denaturation at 94°C for 1 min, annealing at 56°C for 1 min, and extension at 72°C for 1 min, with a final extension time of 5 min at 72°C. PCR products were evaluated by 1% agarose gel electrophoresis [38].

A 40% [containing 2.8 M urea and 16% (vol/vol) formamide] to 60% [containing 4.2 M urea and 24% (vol/vol) formamide] linear DNA denaturing gradient was formed in 8% (wt/vol) polyacrylamide gels. The gels were submerged in 1× TAE buffer. Approximately 30 μl of PCR product was applied to each well and separated by electrophoresis for 16 h at 58°C using a fixed voltage of 60V in the Bio-Rad DCode System (Bio-Rad Laboratories, Inc., Hercules, CA, USA). Gels were stained for 15 min in 1× TAE buffer containing 0.5 μg/ml ethidium bromides, followed by 15 min of rinsing in 1×TAE buffer after electrophoresis. DGGE profile images were digitally recorded using the Molecular Imager Gel Documentation system (Bio-Rad Laboratories, Inc., Hercules, CA, USA) [38].

### DGGE profile analysis

DGGE profile analysis was performed based on the migration distances and intensity of each detected band by GelCompare II software version 6.5 (Applied Maths, Kortrijk, Belgium). Each gel was normalized according to a DGGE standard marker included in all the gels, which was generated using 5 species-specific strains (*Lactobacillus acidophilus* ATCC4356, *Streptococcus mutans* UA159, *Actinomyces naeslundii* 12104, *Streptococcus gordonii* ATCC 10558, and *Streptococcus sanguinis* ATCC10556). The background was subtracted using mathematical algorithms according to the spectral analysis of overall densitometric curves. A minimal profiling setting (1.0%) was used for band searching.

The diversity index of the oral or nasal microbiota was evaluated using the Shannon-Wiener index (*H*) and richness (*S*) based on the following equations:

$$H=\overset{S}{\underset{i=1}{\sum }}pi\ln\;pi$$ (1)

$$E_{H}=\frac{H}{Hmax}=\frac{H}{lns}$$ (2)

Where *p*_*i*_ is the ratio of the intensity of a single band to the total intensity of all bands within the same lane, and *S* is the total number of bands in each lane [39].

Similarities between profiles were analyzed by the Pearson product moment correlation coefficient of similarity [40], using the unweighted pair group method by means of arithmetic averages (UPGMA) [41]. Dendrograms of genetic similarity between samples were calculated.

Principal component analysis (PCA), which was used to calculate the dendrograms of each DGGE gel [42], was performed with Canoco 4.5. By PCA analysis, different data of the detailed DGGE patterns of each sample could be directly analyzed and reduced to one spot in a two dimensional space.

### 16S rRNA gene sequencing

Distinct amplicons from DGGE gels were excised, and transferred to a 200 μl PCR tube containing 10 μl sterile nuclease-free water (Fisher Scientific, Wilmington, USA). After incubating at 4°C overnight, the recovered PCR products were reamplified with the same Bac1 and Bac2 primers lacking the GC clamp. The resulting PCR products were purified and sequenced on an ABI 3730XL automated DNA sequencer (Applied Biosystems, Foster City, CA, USA). Obtained sequences were analyzed using ABI Sequence Analysis 5.2 (Applied Biosystems, Foster City, CA, USA), and determined by using the BLASTN search program at the NCBI Web site and HOMD (Version 10.1;http://www.homd.org/).16S rRNA sequence Identification. Sequences that had 98% higher similarity were considered positively identify their respective taxon.

### Statistical analysis

Fisher’s exact test was used to compare the gender distribution and the frequencies of the sequenced bands representing distribution of major bacteria between the two groups. Nonparametric Mann-Whitney U tests were used for continuous dependent variables. The results for some continuous variables are presented as mean ± standard deviation (SD), range, and 95% confidence interval (CI). The level of significance was set at p<0.05. For all comparisons, the p-values are presented, even if they are not significant. All statistical analyses were performed with SPSS software version 19.0 (SPSS Inc., Chicago, IBM).

## Results

### Study subjects characteristics

Twelve radiation caries-free (RCF) patients and nine patients exhibiting radiation caries (RC) participated in this study. The study subjects had completed their RT 12 to 36 months before enrolling in this study. There were no significant differences in gender distribution, mean age, bilateral size of parotid gland, and V40 of the two groups (Table 2).

**Table 2:** Clinical features of the subject population.

	RCF group		RC group	
	n = 12		n = 9	
	Mean±SD	Range	Mean±SD	Range
**Age(years)** ^**a**^	45.4±14.1	(22–68)	52.3±11.0	(34–68)
**Gender** ^**b**^	M = 58.3%	M = 7	M = 66.7%	M = 5
	F = 41.6%	F = 5	F = 33.3%	F = 4
**Bilateral size of parotid gland L/R(cm** ^**3**^)^**a**^	44.31±18.63	(25.23–76.30)	43.92±15.63	(26.59–75.06)
**Dosage cGy** ^**a**^	7033±115	(7000–7400)	7088±174	(7000–7400)
**V40(%)** ^**a**^	33.41±4.82	(23.51–40.72)	36.40±6.37	(24.79–42.76)

^a^Independent sample nonparametric Mann-Whitney U test was used;

^b^Fisher’s Exact test was used.

Despite variation in volume of the parotid gland between individuals, they were all within the normal range (25.8–120cm^3^) [43, 44]. It has been reported that V40 should be less than 33% for complete saliva production recovery after 24 months [32]. In our cohort, eleven out of twelve RCF subjects had V40 over 33%, while eight out of nine subjects were in the RC group.

### Oral health information

The general oral health of the study subjects is presented in Table 3. There are significant differences in the plaque index between the two groups (p = 0.038). None of the study subjects had clinical use of fluorine products and artificial saliva. The use of fluoride toothpaste was investigated through a questionnaire when the study subjects enrolling in the study. None of them had used fluoride mouthrinse (Table 4). Furthermore, no special food preferences were observed.

**Table 3:** Oral health information of RCF and RC subjects.

	RCF group n = 12		RC group n = 9	
	Mean±SD	Range	Mean±SD	Range
**Newly DMFT**	0	0	12.0±6.42	(6–21)
**Plaque index** ^**a**^	1.06±0.42	(0.35–1.59)	1.52±0.49	(0.79–2.09)*
**Frequency of teeth brushing(times/day)**	2.08±0.42	(2–3)	1.78±0.44	(1–2)
**Clinical application of Fluorine production**	No	-	No	-
**Use of artificial saliva**	No	-	No	-
**Preference of food**	No	-	No	-

^a^Independent sample nonparametric Mann-Whitney U test was used.

**Table 4:** Use of Toothpaste of RCF and RC subjects.

Fluoride type/Concentration	Radiation caries-free group(n)	Radiation caries group(n)
Sodium fluoride /0.06%-0.14%	10	8
Sodium fluoride /0	2	1

### Salivary function

The stimulated saliva flow rate, saliva pH value, and buffering capacity are showed in Table 5. Our data show that the stimulated saliva flow rate in the RCF group is far lower than the lowest normal standard, reported to be 2.0 ml/min [27, 45]. Although the mean stimulated saliva flow rate was higher in the RCF group than in the RC group, there was no statistically significant difference between the two groups. The pH value of saliva ranged from 6.77 to 7.41, which is in line with reported normal salivary pH range (5.3 to 7.8) [46]. Our results show that the pH and buffering capacity of saliva from both the RCF and RC groups fall within the normal range.

**Table 5:** Salivary function of subjects.

Subjects	Stimulated saliva flow rate(ml/min)^a^		Saliva pH^a^		Buffering capacity No. of patients (%)	
	Mean±SD	Range	Mean±SD	Range	low	high
**RCF group n = 12**	0.98±0.42	(0.6–2)	7.02±0.20	(6.80–7.41)	0	12(100)
**RC group n = 9**	0.66±0.27	(0.05–0.9)	6.96±0.19	(6.77–7.40)	0	9(100)

^a^Independent sample nonparametric Mann-Whitney U test was used.

### Clustering of DGGE profiles

**Fig 1:**
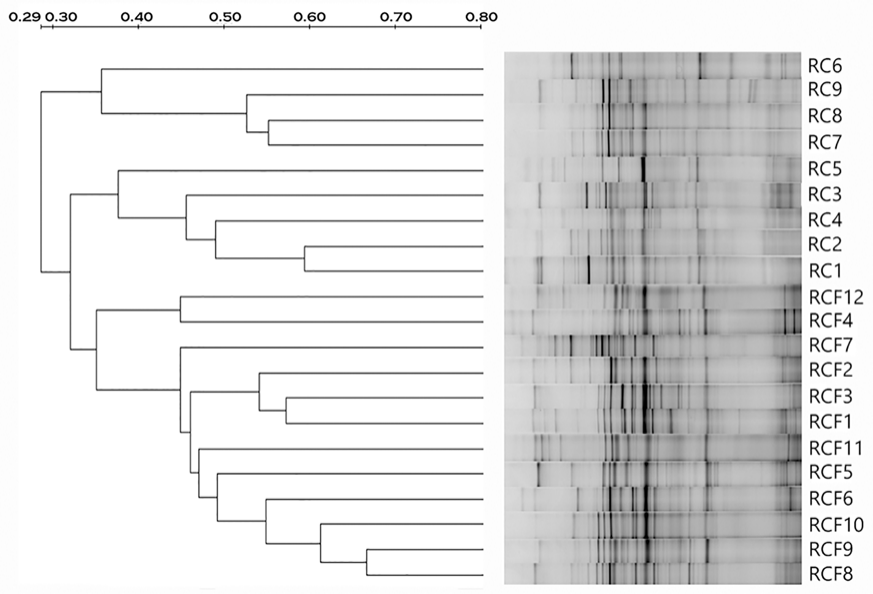
Clustering analysis results of all DGGE profiles demonstrated graphically as an UPGMA dendrogram. Percentage similarity is shown on the scale above the dendrogram.

We compared the DGGE profiles 16S ribosomal genes amplified from the saliva of RCF and RC subjects. We used the Pearson product moment correlation coefficient and dendrograms to analysis similarity between salivary microbes in both groups (Fig 1). The dendrogram resulting from Dice cluster analysis of saliva samples show three distinct clusters with four RC subjects in cluster I (36% similarity), the remainder of the RC group in cluster II (38% similarity), and all the RCF subjects in cluster III (35% similarity).

### Analysis of DGGE banding patterns

**Fig 2:**
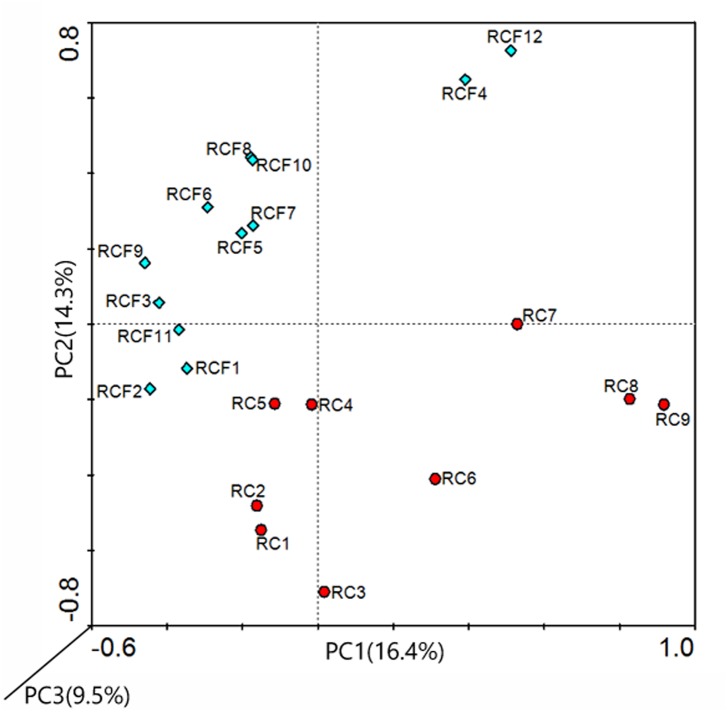
PCA analysis of samples. Principal components were calculated for each lane of each DGGE gel with group-specific 16S rDNA fragments. RCF group (blue dots); RC group (red dots).

DGGE banding patterns were examined by computer-aided analyses based on densitometric curves. The number of peaks and their intensity in the denstiometric curve of the gel reflect the number and relative abundance of the predominant 16S rRNA gene sequences. PCA is classified as dimensioning mechanics among grouping mechanics. It is worth noting that all DGGE patterns available were involved to perform PCA analysis. Basing on the relatedness, it produces two-dimensional plots where the entries are spread. Additionally, the construction is more subjective than dendrogram. In our study, a clear separation between spots and an evenly distributed spots were seen, and a low and almost mean percentage of total variation was revealed by the three principal components (PC) (only PC 1 and PC 2 were adopted to the analysis) (Fig 2).

The diversity indices (*H*, *S* and *E*_*H*_) of microbial samples from saliva are presented in Table 6. No significant differences in diversity indices were found in the stimulated saliva samples. Although the RCF group showed higher Shannon-Wiener index and richness than the RC group, we did not detect statistical differences in *H*, *S* and *E*_*H*_ between the two groups.

**Table 6:** Comparison of the diversity indices calculated from DGGE profiles.

Diversity Indices^a^	RCF group	RC group
***H***	2.54±0.16	2.52±0.14
***S***	12.90±2.16	12.49±1.78
***E***_***H***_	16.49±3.01	16.12±2.63

*H*: Shannon-Wiener index, *S*: Richness, *E*_*H*_: Evenness;

^a^Independent sample nonparametric Mann-Whitney U test was used.

### Sequence analysis

**Fig 3:**
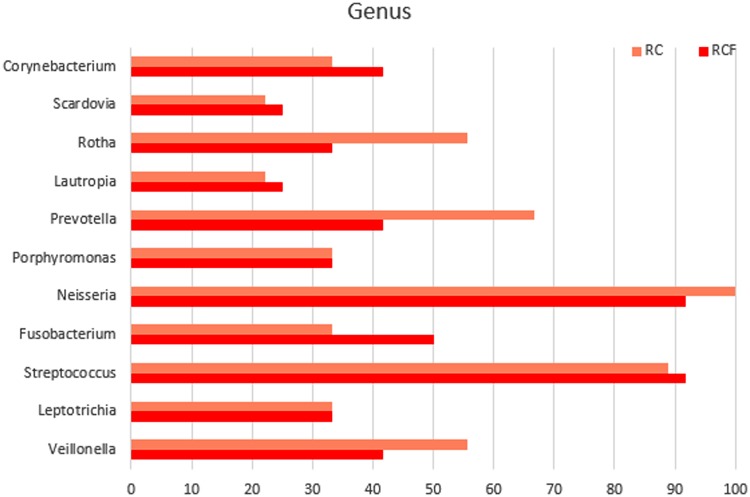
Distribution of taxonomy profiles according to 16S rDNA V3–V5 region sequence analysis.

In total, 175 distinct amplicons were excised from the DGGE gels. Eleven genera were identified (Fig 3), and the frequencies of their occurrence in different types of samples analyzed between groups. *Streptococcus* and *Neisseria* had the highest distribution. *Scardovia* and *Lautropia* were present at very low distribution (about 20%). Similar distributions were detected in the RC group. Among all genera, there were no ‘gender-specific’ (present in one gender but absent in the other) or ‘gender-associated’ (differentially distributed yet present in both gender) genera detected (data not shown). No ‘radiation-caries-specific’ genera (present only in either the RCF or the RC populations) was detected either.

### Microbial cultivation results

**Fig 4:**
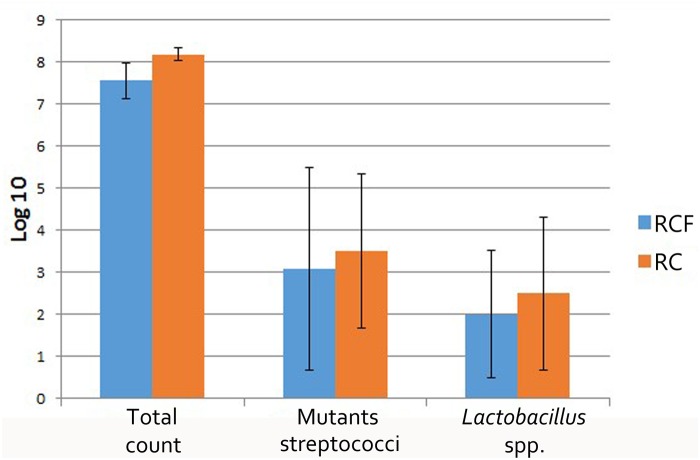
Mean±SD log_10_ number of microorganism in subjects with RCF (n = 12)and RC (n = 9).

The log CFU counts of total cultured bacteria, LB and MS also presented in Fig 4. Although the average log CFU of total cultured bacteria, LB and MS in the RCF group is lower than that from the RC group, there was no significant difference between these two groups.

## Discussion

We initially hypothesized that salivary function and salivary microbiota are potentially related with the absence of radiation caries one year or more after RT. To our knowledge, this is the first paper to report on these characteristics in RCF irradiated NPC patients. Most existing research focused on RC patients within the first year post-RT, during which they have the highest risk of suffering from radiation caries [14, 28]. In this study, all participants have completed IMRT for at least one year.

In this study, salivary function was measured through stimulated flow rate, saliva pH and buffering capacity [23]. Previous researches has demonstrated that IMRT-treated patients still commonly experienced loss of salivary function [24]. The mean stimulated salivary flow rate in healthy control groups has been previously reported to be above 2.0ml/min [28, 45]. However, in our study, the mean stimulated salivary flow rate in the RCF group was significant lower than normal, as was the flow rate in the RC group. This findings is in agreement with several publications which have reported that the mean stimulated saliva flow rate of irradiated patients do not recover to their baseline even one or two years after IMRT [23, 47].

Notably, we did not detect any significant decreases in pH value and buffering capacity of the stimulated saliva when compared with normal values. The pH value of stimulated saliva in our study ranged from 6.77 to 7.41, which is in line with reported ‘normal’ pH between 5.3 and 7.8 [46]. It is interesting to note that, some researchers have reported a decrease in salivary pH from about 7.0 to 5.0 in combination with a loss in buffering capacity immediately after RT [18, 48, 49]. As salivary flow gradually increases with time after RT, the secreted saliva also became less acidic. The mean salivary pH improves to 6.9 after year 1 and recovered to 7.2 at 2 years after IMRT. Additionally, the proportion of RT patients showing saliva with medium-to-high buffering capacity increases to 70.6% at 1 year post-RT and to 85.7% at 2 years post-RT [23]. Hence, a new buffering system may become established approximately one year after IMRT. However, since the salivary flow rate is not yet recovered in our study subjects, who are 12–36 months post-RT, it is possible to conclude from the given data that salivary function in RCF individuals is still damaged. A similar conclusion may be drawn from the RC group.

It has been reported that the oral microbiota of irradiated patients intrinsically changes with their salivary function[50]. The diversity index and major species were analyzed to characterize the oral microbiota. The Shannon-Wiener index (*H*), richness (*S*) and evenness (*E*_*H*_) values characterizing salivary microbes from the RCF group and RC groups showed no statistical differences, suggesting that similar microbial diversity is found in these two groups. However, these data only compare the diversity between a caries-free group and a caries bearing group. Whether caries-bearing individuals exhibit higher [51, 52] or lower [53, 54] microbial diversity than caries-free subjects is still debated. Our results conflicted with previously published reports, possibly because all the participants in those studies had not treated by RT and had healthy salivary function. However, our data only showed a weak correlation between oral microbial diversity with the absence of radiation caries.

In our study, eleven genera (*Streptococcus*, *Neisseria*, *Scardovia*, *Porphyromonas*, *Fusobacteria*, *Lautropia*, *Veillonella*, *Capnocytophaga*, *Rithia*, *Leptotrichia* and *Prevotella*) of microbes were found and *Streptococcus* spp. and *Neisseria* spp. represented over 80% of samples. Likewise, a similar distribution has been shown in previous surveys [3, 31, 55]. *Veillonella*, *Capnocytophaga*, *Neisseria*, *Rithia*, *Leptotrichia* and *Prevotella* were detected in both irradiated groups, which matched the results of previous studies [3, 31]. Other genera, such as *Granulicatella* and *Gemella*, were detected by Hu et al. partly because they had planted in oral cavity before RT began. *Porphyromonas* and *Fusobacteria* were observed in both groups partly because they had been shown to be healthy oral genera in previous studies [56]. *Scardovia* has been shown to be significantly increased in cavitated dentin lesions of children [57]. It is reasonable to expect factors such as age, diet and life-style to affect oral microbial community structures [58]. In spite of differences between individuals, there were no statistical differences in the distribution of taxa in both the RCF and RC groups. Alternatively, a similar composition of major species may explain the same diversity index found in RCF and RC subjects, due in part to the same destruction of salivary function brought on by radiotherapy [2, 59]. However, contrary to our hypothesis, our data showed a weak correlation between major microbial species with the absence of radiation caries.

In this study, we also adapted traditional method to culture LB and MS from stimulated saliva. Interestingly, the number of MS we found is consistent with previously published figures, while the number of LB is not in agreement [28, 60]. The log CFU counts of LB were lower than the previously reported 4 or 5. However, as discussed above, most of the previous reports focused on irradiated patients within the first 6 months after ordinary RT [27, 28] or within the first year after ordinary RT[14]. From the sixth month after RT, LB partly returns to its baseline composition [12]. In this study, we used both traditional microbial culture as well as molecular biology techniques to minimize the bias in each assay. However, the limitation of DGGE should not be ignored and levels of certain microbes in the samples may be below the detection limit of PCR (10^2^ cells) [61]or DGGE (10^3^ cells) [62]. The visible bands are considered to be from species that represent more than 1% of the sample microbiota [63]. Our data showed that the number of LB was below 10^3^. This may explain why LB was not detected when microbial diversity was investigated with DGGE. In agreement with previous reports, LB was absent in studies employing the Human Oral Microbe Identification Microarray (HOMIM) [52, 64]. As the same quantity of LB and MS were detected in both the RCF and RC groups, we observed a weak correlation with the absence of radiation caries, and the role of LB and MS in the process of developing radiation caries may have been overestimated.

In our study, we found lower levels of plaque index (PL) in RCF group participants than RC group participants (p = 0.038). Since PL is a direct assessment of the oral hygiene level, a significant difference in PL indicates that the RCF group has better oral hygiene than the RC group. However, the cross-section nature of this project did not allow for any causative statements. Therefore, the question of whether bad oral hygiene was a causative factor in foregoes radiation caries development or whether it is a result of radiation caries could not be addressed.

### Conclusion

In conclusion, in this study we find no clear correlation between the characteristics of patient salivary microbiota and radiation caries one year after IMRT. Our results suggest that salivary function in irradiated patients does not recovered fully after 12–36 months, but the pH value and buffering capacity of saliva return to normal after one year or more following IMRT. However, population-based longitudinal studies are required to reveal the factors for the absence of radiation caries.
